# Impact of Genetic Selection for Increased Cattle Resistance to Bovine Tuberculosis on Disease Transmission Dynamics

**DOI:** 10.3389/fvets.2018.00237

**Published:** 2018-10-01

**Authors:** Kethusegile Raphaka, Enrique Sánchez-Molano, Smaragda Tsairidou, Osvaldo Anacleto, Elizabeth Janet Glass, John Arthur Woolliams, Andrea Doeschl-Wilson, Georgios Banos

**Affiliations:** ^1^The Roslin Institute and Royal (Dick) School of Veterinary Studies, University of Edinburgh, Edinburgh, United Kingdom; ^2^Department of Agricultural Research, Gaborone, Botswana; ^3^Instituto de Ciências Matemáticas e de Computação, Universidade de São Paulo, São Carlos, Brazil; ^4^Scotland's Rural College, Edinburgh, United Kingdom

**Keywords:** bovine tuberculosis, resistance, susceptibility, epidemiological model, genetic selection, prevalence

## Abstract

Bovine tuberculosis (bTB) poses a challenge to animal health and welfare worldwide. Presence of genetic variation in host resistance to *Mycobacterium bovis* infection makes the trait amenable to improvement with genetic selection. Genetic evaluations for resistance to infection in dairy cattle are currently available in the United Kingdom (UK), enabling genetic selection of more resistant animals. However, the extent to which genetic selection could contribute to bTB eradication is unknown. The objective of this study was to quantify the impact of genetic selection for bTB resistance on cattle-to-cattle disease transmission dynamics and prevalence by developing a stochastic genetic epidemiological model. The model was used to implement genetic selection in a simulated cattle population. The model considered various levels of selection intensity over 20 generations assuming genetic heterogeneity in host resistance to infection. Our model attempted to represent the dairy cattle population structure and current bTB control strategies in the UK, and was informed by genetic and epidemiological parameters inferred from data collected from UK bTB infected dairy herds. The risk of a bTB breakdown was modeled as the percentage of herds where initially infected cows (index cases) generated secondary cases by infecting herd-mates. The model predicted that this risk would be reduced by half after 4, 6, 9, and 15 generations for selection intensities corresponding to genetic selection of the 10, 25, 50, and 70% most resistant sires, respectively. In herds undergoing bTB breakdowns, genetic selection reduced the severity of breakdowns over generations by reducing both the percentage of secondary cases and the duration over which new secondary cases were detected. Selection of the 10, 25, 50, and 70% most resistant sires reduced the percentage of secondary cases to <1% in 4, 5, 7, and 11 generations, respectively. Similarly, the proportion of long breakdowns (breakdowns in which secondary cases were detected for more than 365 days) was reduced by half in 2, 2, 3, and 4 generations, respectively. Collectively, results suggest that genetic selection could be a viable tool that can complement existing management and surveillance methods to control and ultimately eradicate bTB.

## Introduction

Bovine tuberculosis (bTB) is an infectious zoonotic disease of cattle caused by *Mycobacterium bovis* (*M. bovis*) that is endemic in many parts of the world (1). Notably, bTB continues to be a challenge in the United Kingdom (UK) despite a national eradication programme being in place for over five decades (2). In the UK, bTB control is mainly based on the culling of cattle that react positively to the single intradermal comparative cervical tuberculin test, commonly known as the skin test. When at least one positive reactor to the skin test is detected in a herd during routine testing, a “breakdown” status is declared, and animal movement restrictions are imposed on that herd. The herd is then systematically tested every 2 months and animals reacting positively to the skin test are sent to slaughter. When all animals test negative to two consecutive tests the breakdown officially ends and the herd re-enters routine surveillance (3).

In addition to herds being subjected to compulsory regular testing, other control measures are applied in relation to bio-security (2, 4). However, so far, the existing control strategies have proven insufficient to eradicate the disease. This may be partially attributed to the low sensitivity of the skin test, potentially leading to undetected infected animals that contribute to the recurrence of breakdowns (5). Another contributing factor is the existence of wildlife reservoirs of *M. bovis* (for example, the Eurasian badger in the UK) (6). The problem persists and there is no clear evidence for a decline (7), despite the UK government spending over £175 million annually in the control of the disease (8). While Scotland was declared officially bTB free (OTF) in 2009, the governments of England and Wales have set a goal to become OTF by 2038 (4, 9). Thus, genetic selection for increased resistance of cattle to bTB may provide a potential complementary strategy (10) to achieve this goal.

Quantitative genetic studies have shown that there is genetic variation in cattle resistance to bTB (11–15). Therefore, it would be feasible to reduce disease prevalence and breakdown severity through selectively breeding for enhanced host resistance to the disease. In the UK, genetic evaluations of individual dairy cattle for resistance to bTB have been available since 2016. Availability of genetic evaluations enables the bovine industry to select sires based on their inherent capacity to produce more resistant progeny (16). However, before embarking on intense selection for enhanced resistance to bTB, it is important to understand the impact of such a selection process on disease risk and prevalence (17).

Genetic epidemiological models have been used to evaluate the role of genetic selection in populations undergoing an epidemic (17–19). Such models have been applied to a variety of diseases in farm animals including sea lice infection in the Atlantic salmon (20), bacterial (21, 22), and nematode (23) infestations in sheep, and Marek's disease in chickens (24). These studies estimated the impact of host genetic variation and genetic selection for increased host resistance on disease prevalence and spread. Several epidemiological models specific to bTB in cattle have been proposed (5, 25–31). None of them, however, has accounted for genetic variation in host resistance or considered genetic selection as a potential control option. In the present study, we propose an epidemiological model which, unlike previous models for bTB, incorporates genetic variation of disease resistance in the host, and models genetic selection.

Disease progression in previous epidemiological bTB models has been typically assumed to follow transition from the state of susceptible (*S*) to exposed (*E*), to test-sensitive (diagnosable; *T*), and finally to Infectious (*I*; *SETI* model). Typically, a susceptible animal becomes infectious only after going through the exposed and test-sensitive states (5, 27, 28, 30, 31). Pathogen transmission in the *SETI* model is such that infected animals that are test-sensitive and react positively to the skin test are removed before they become infectious. If this is the case, identification of infected animals through frequent comprehensive testing and immediate removal of test-positive animals as being currently carried out in the UK should substantially reduce bTB prevalence. However, given the current gap of knowledge about the relationship between *M. bovis* excretion and skin test response, and considering the persistence and general increase in bTB incidence over the past decade in the UK (7), other models of disease transmission dynamics need to be explored.

In the present study we considered a *SEIT* model where an animal becomes infectious (*I*) before infection can be detected by the skin test (*T*). This model implies that infected cattle may become infectious before they can be diagnosed and removed. Compared to the *SETI* model, *SEIT* represents the “worst case” scenario in terms of bTB transmission. The model follows the suggestion that all tuberculous cattle with lesions, particularly in the respiratory tract, should be considered as potential excretors of *M. bovis*, thus constituting sources of infection for other animals both within and across herds (32, 33).

The aim of the present study was to determine the impact of genetic selection for enhanced host resistance to bTB on cattle-to-cattle transmission dynamics and bTB prevalence using a *SEIT* epidemiological model.

## Material and methods

The impact of selection for increased resistance to bTB on the risk and severity of bTB breakdowns were investigated using a simulated, genetically heterogeneous cattle population. The proposed genetic epidemiological model was designed to simulate *M. bovis* infection dynamics in closed herds within the current UK bTB testing policy, firstly in the absence of selection and secondly following genetic selection for enhanced host resistance (reduced susceptibility) over 20 generations, with different selection intensities.

### Simulated populations

Non-overlapping generations of a dairy cattle population (*N* = 20,000) were generated comprising 50% males and 50% females. A founder generation was created, where sires and dams were randomly chosen and mated to create the base population. This base population was generated assuming a sire-to-offspring ratio of 1:50, thus being consistent with the national policy in reporting genetic evaluations for bTB in the UK (R. Mrode, personal communication, 2017). Large half-sib families were thus created, reflecting a realistic dairy cattle population structure where, with the extensive use of artificial insemination, sires tend to have large numbers of progeny (daughters). Given that genetic selection of the best sires is the key component of selective breeding programmes in dairy cattle, selection was carried out based on estimated breeding values of sires generated as outlined below. This is also consistent with the current industry practice to only consider sire bTB genetic evaluations in selection.

### Incorporating genetic variation in host susceptibility

Cattle susceptibility to bTB was modeled as a polygenic trait consistent with an infinitesimal model assuming presence of many loci each with a small additive effect on the trait (15, 34). More specifically, genetic variation for susceptibility was assumed to follow a normal distribution in the log scale, since previous studies suggested that disease traits are usually skewed (20, 35–37) and a log transformation is commonly used to achieve data normality (38). Considering that genetic evaluation methods may not capture all the additive genetic variance ($\sigma_{a}^{2}$) associated with a trait, therefore, both the true genetic value of an individual (TBV) for susceptibility and the corresponding estimated breeding value (EBV) were simulated drawing from normal distributions $N(0,\;\sigma_{a}^{2}$) and $N(0,\;r^{2}\sigma_{a}^{2}$), respectively, where *r* was the accuracy of the estimate. Thus, in the founder population, TBVs and EBVs were simulated from a multivariate normal distribution *MVN*(0, **G**), where **G** corresponded to the genetic variance-covariance matrix. The covariance between TBVs and EBVs was derived as *cov*_*TBV, EBV*_ = r$*\sqrt{\sigma_{a}^{2}}*\sqrt{\sigma_{a}^{2}r^{2}}$. An additional term, the prediction error (PE) for each animal was computed as the difference between TBV and EBV.

In further generations, TBVs of the offspring of two selected animals were equal to the average TBV of the parents plus an individual Mendelian sampling (MS) term reflecting the random sampling and combination of parental alleles. This latter term followed a normal distribution$N(0,\;0.5(1-\bar{F})\sigma_{a}^{2}$), where $\bar{F}$ corresponded to the average inbreeding coefficient of the parents. In a similar way, the TBVs of the offspring were decomposed into EBV and PE, both being computed as the average of the respective parental values plus the corresponding MS terms, which were now drawn from normal distributions $N(0,\;0.5(1-\bar{F})\sigma_{EBV}^{2}$) and $N(0,\;0.5(1-\bar{F})\sigma_{PE}^{2}$), respectively. Therefore, simulated TBVs, EBVs, and PEs were computed for each offspring as:

$$\begin{array}{l} EBV_{offspring}={\bar{EBV}}_{parents}+MS_{EBV}\\ PE_{offspring}={\bar{PE}}_{parents}+MS_{PE}\\ TBV_{offspring}=EBV_{offspring}+PE_{offspring} \end{array}$$

In all generations, environmental effects were generated from a normal distribution $N(0,\;\sigma_{e}^{2}$ corresponded to the environmental variance and was kept constant through all generations. Finally, the individual phenotypic value for underlying susceptibility to bTB i.e., g_*i*_ of each individual animal *i* was computed as the sum of the animal's TBV plus the corresponding environmental effect E, i.e., *g*_*i*_ = *TBV*_*i*_ + *E*_*i*_.

### Distribution of animals into individual herds

Currently, genetic evaluations for bTB in the UK assess the resistance of sires based on disease incidence of their daughters as described in Banos et al. (39). Therefore, breakdowns were simulated here based only on female offspring produced in each generation; the latter corresponds to 2–4 years in dairy cattle. A pool of selected sires was created, and female offspring were randomly allocated into 100 herds comprising 100 individuals each. Every selected sire contributed at least one daughter into one herd. Breakdowns were then simulated within each herd as outlined below.

### The epidemic within herd transmission model

A stochastic within-herd bTB transmission model was developed to simulate bTB spread in each herd and provide estimates of severity and duration of bTB breakdowns (Supplementary Figure 1). In particular, a compartmental *SEIT* model was assumed in which susceptible cows progress between the four infection states: (1) Susceptible state (*S*), where the animal is not infected but susceptible to infection; (2) Exposed state (*E*), where the animal is infected but not infectious and is undetectable by the skin test; (3) Infectious state (*I*), where the animal is able to infect others but is still undetectable by the skin test; (4) Test-sensitive state (*T*), where the infectious animal is now detectable by the skin test. Furthermore, the model incorporated the current UK policy of a 60 days routine skin test performed on all animals following the onset of a breakdown. At the specific test-days, infected animals at detectable state *T* may be diagnosed as reactors assuming a test sensitivity of Ω. Cows that reacted positively to the skin test were removed from the herd, in line with the UK official test-and-cull procedure (Supplementary Figure 1).

Infection (transition from state *S* to *E*) was modeled as a Poisson distribution process with time dependent average infection rate λ(*t*) = α + β(*I*(*t*) + *T*(*t*)), where *I*(*t*) and *T*(*t*) were the number of animals in the herd at the *I* and *T* states at time *t*, respectively, and the parameters α and β represented transmission coefficients for external sources of infection (aggregate of all potential sources of external infection including wildlife, infected move-in cattle and infected cattle from contiguous farms) and for within-herd cattle-to-cattle transmission, respectively (Supplementary Figure 1) (30, 31). A density dependent mode of transmission was assumed as herd size is known to be correlated to bTB incidence and persistence (40–42). Progression of infected cows from *E* to *I* state and from *I* to *T* state occurred at average rates σ and γ, respectively (Supplementary Figure 1).

Individual variation in susceptibility was incorporated into the model through each individual's log-normally distributed susceptibility phenotype calculated as outlined above. The individual infection rate of individual *i* at time *t* was then defined as $\lambda_{i}\left(t\right)=e^{g_{i}}\left(\alpha +\;\beta \left(I\left(t\right)+T\left(t\right)\right)\right)$, where *g*_*i*_ refers to the normally distributed susceptibility value specified by the genetic model above. In contrast to the population averages for α, β σ, and γ, which were kept constant over successive generations, the average susceptibility *g* changed over generations because of genetic selection.

To generate a sufficient number of herds experiencing breakdowns in the first generation, the epidemic in each herd was started by two randomly chosen infectious individuals in state *I*, termed “index cases.” Two individuals were chosen here instead of one to ensure that breakdowns did not die out within the first 60 days of duration. This editing step allowed us to generate enough data to test the various genetic selection practices described below.

Disease progression within each herd was then simulated as a series of random independent events representing the transition of an animal between two successive states in the compartmental *SEIT* model. The time to the next event (inter-event time), the corresponding event type (for example, transition from *S* to *E*), and the corresponding individual experiencing the transition were determined using Gillespie's direct algorithm adapted to heterogeneous populations as outlined in Lipschutz-Powell et al. (35).

Possible events in our model were the infection of a susceptible animal (transition from *S* to *E*), an exposed animal becoming infectious (transition from *E* to *I*), an infectious animal becoming test-sensitive (transition from *I* to *T*) and a test-sensitive animal being removed from the herd after testing positive to the skin test (transition from *T* to *R*). However, the latter event was modeled separately at time intervals of 60 days according to the official skin test schedule. For the other events the inter-event time was sampled from an exponential distribution with rate equal to the sum of all process rates calculated as $R_{total}=\overset{N_{S}}{\underset{i=1}{\sum }}e^{g_{i}}\left(\alpha +\beta \left(I+T\right)\right)+\sigma N_{E}+\gamma N_{I},\;$where *Nx* is the total number of animals in each *x* state within the herd. In other words, the time to the next event was estimated as −ln(*y*)/*R*_*total*_, where *y* ~ *U* (0, 1). The specific event type *e* that occurs at that particular time was sampled by drawing a random variable from a distinct distribution with probability $\frac{{p\left(e\right)=R}_{e}}{R_{total}}$. *R*_*e*_ is the rate of occurrence of the specific event. The individual in the particular event was then chosen randomly, and in the case of infection (*S* to *E*) it was weighted by the individual's susceptibility phenotype.

In line with the current bTB control strategy, the epidemic in each herd was simulated until the end of a bTB breakdown, defined by two consecutive negative skin tests for all herd members (3). During the epidemic, the number of individuals in each disease state together with the corresponding times was recorded, and based on these, the total number of reactors and the duration of each epidemic (i.e., the time from beginning to end of a breakdown) were derived.

### Model parameterization and validation

Input parameters for the epidemiological bTB model illustrated in Supplementary Figure 1 were based on real field data used for national genetic evaluations for bTB in the UK. These data consisted of 1,210,652 cow records from 10,589 herds where breakdowns had been declared between the years 2000 and 2014. The mean number of animals per herd in the dataset was 114, and the recorded number of infected animals referred to reactors diagnosed by the skin test. Based on the latest bTB epidemiological study in the UK (31) the value of the external rate of infection α in the simulation (Supplementary Figure 1) was set to 5 × 10^−7^ days^−1^. Furthermore, a skin test sensitivity (Ω) of 0.60 was used as in Banos et al. (39), which is the value considered in the current official UK genetic evaluation for bTB resistance. To determine the remaining parameter values of the *SEIT* model (β, σ, γ, as well as genetic and environmental variances for underlying susceptibility), multiple parameter combinations were tested and the corresponding model output was compared to the following characteristics derived from analyzing the field data: mean percentage of skin-test reactors per breakdown (8.5%), mean duration of breakdown from official onset to end (366 days), and genetic variance (0.0032) and heritability (0.10) of the observed bTB phenotype indicating presence (reactor) or absence (non-reactor) of bTB. We derived these estimates from the analysis of the above-mentioned field data using the model described in Banos et al. (39).

The bTB susceptibility phenotype *g* in the *SEIT* model (Supplementary Figure 1) corresponds to the underlying scale of the binary presence or absence of the disease trait in the data analyses (39) (observed scale). To make the model results concordant with the observed scale, a range of different genetic and environmental variance estimates for the underlying scale in the base population were explored and the corresponding heritability and genetic variance estimates on the observed scale were calculated. The final genetic and environmental variances chosen for the simulated data on the observed scale and used to generate the base population were those that were closest to the real field data estimates on the observed scale.

In order to study the impact of variation in epidemiological parameters on disease epidemic and genetic selection, two additional simulation scenarios were run, one assuming a 10-fold increase in the rate of external infection (α = 5 × 10^−6^ days^−1^) and another considering a lower sensitivity of the skin test (Ω = 0.30); the latter is similar to the lower credible interval obtained in the meta-analysis of skin-test sensitivity by Nuñez-Garcia et al. (43).

### Genetic selection process and impact

Firstly, epidemics were simulated for 20 generations without any genetic selection (100% of sires used for breeding) in order to establish the baseline of bTB transmission dynamics. Subsequently, truncation selection of genetically resistant sires was simulated for 20 generations. Sires were selected for breeding based on their underlying susceptibility EBVs. Different levels of selection intensity were explored by selecting the 10, 25, 50, and 70% most resistant (least susceptible) sires. These reflect different potential selection strategies against the disease. Selected sires were randomly mated with cows. Dams were randomly selected in each generation. Population size and sex ratios were kept constant across generations. The female offspring of these sires then formed the next generation of individuals for which bTB epidemics were simulated.

The impact of genetic selection on bTB prevalence was assessed in each generation by estimating the mean underlying susceptibility to *M. bovis* infection in the population as well as the risk and severity of breakdowns. A breakdown was assumed to have occurred when at least one secondary case was produced from the index cases within a herd. Otherwise, in the absence of a secondary case a “no breakdown” was declared and duration equal to 0 days was assigned. Therefore, the risk of a breakdown (probability of a breakdown occurring) was defined as the proportion of simulated epidemics that resulted in at least one secondary case (infected cow other than the index cases that seeded the epidemic). The severity of a breakdown was then assessed by estimating the percentage of secondary cases and the duration of their occurrence within the breakdown (duration of secondary cases). Breakdowns were categorized as mild, moderate, and severe based on mean percentage of secondary cases being less or equal to 3% (only 1 secondary case), 3–10%, and above 10% (10% equating 50% of breakdowns in the distribution) respectively. Breakdowns were also categorized as short, medium and long depending on whether the duration of secondary cases was less than or equal to 180 days, between 180 and 365 days, and above 365 days, respectively.

Finally, to assess the impact of the *SEIT* model assumption that animals become first infectious and then test-sensitive, the same simulations were run separately assuming a *SETI* epidemiological model. In the latter, infected animals were test-sensitive, hence detectable, before they became infectious. The same parameters were used as for the *SEIT* model.

In all cases, each selection scenario reflecting one of the four selection intensities described above was replicated 50 times. Results were averaged across all herds and replicates for each generation.

## Results

### Parameter values and model fit to real data

**Figure 1:**
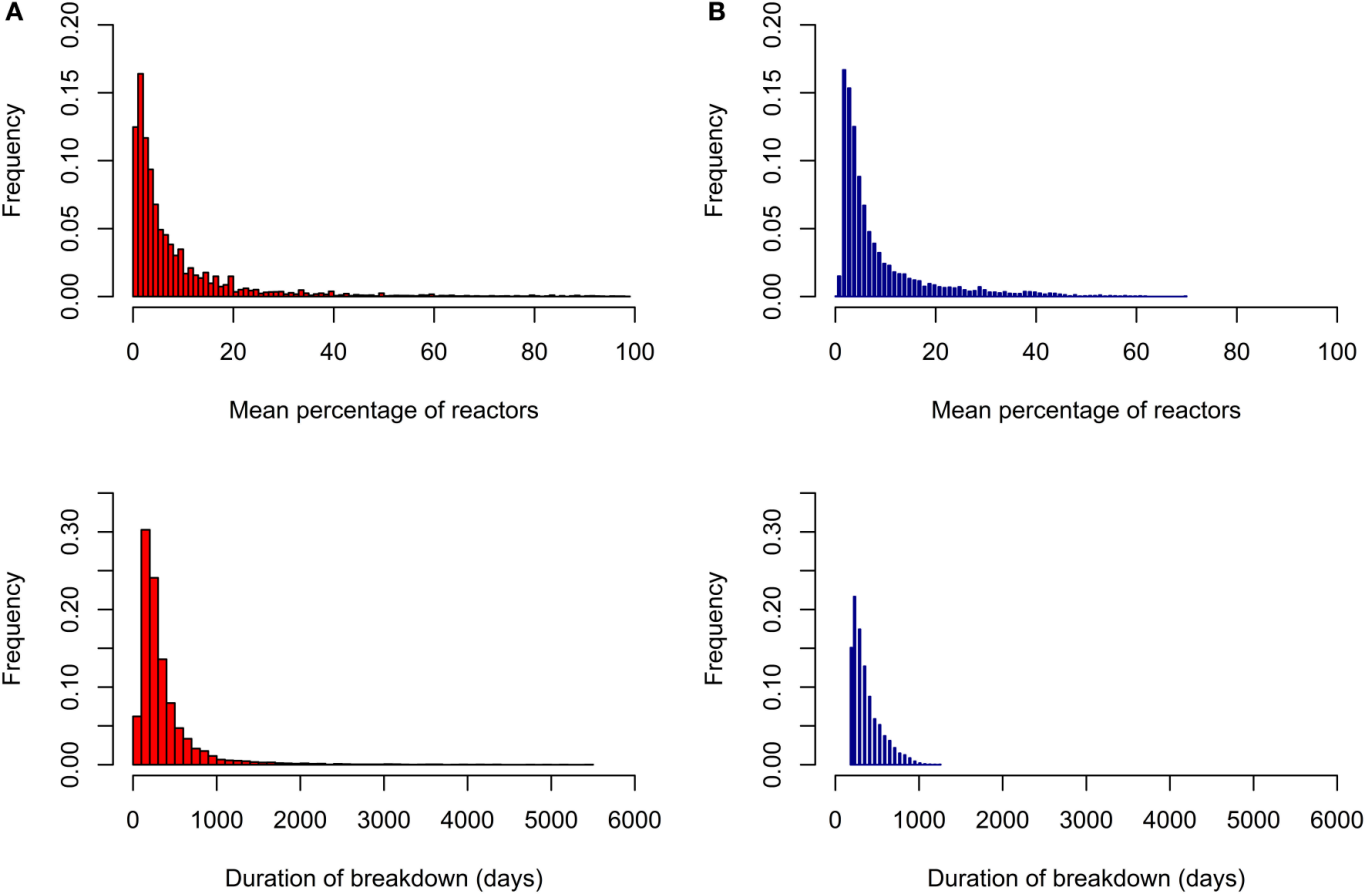
Distribution of percentage of reactors to the skin test per breakdown and duration of breakdown. Results from real data are given in red **(A)** and from simulated data in blue **(B)**.

Parameter values were identified to ensure that simulated and real bTB breakdowns shared similar characteristics with respect to the distributions of mean percentage of reactors per breakdown, total duration of breakdown, genetic and phenotypic variance and heritability of susceptibility on the observed scale (Figure 1; Table 1). The distributions of both the mean percentage of reactors per breakdown and the total duration of breakdown were more long-tailed in real data compared to simulated data (Figure 1), probably because real data were affected by more extreme and unpredictable environmental conditions than those modeled in the simulation. Significant correlations (*p* < 0.001) were found between mean percentage of infected individuals per breakdown and mean duration of breakdown in both datasets; however, the correlation was smaller in real data (0.43) than in simulated data (0.85), for the same reason as stated above.

**Table 1:** Epidemiological and genetic parameters of bovine tuberculosis in simulated and real (field) data.

	**Simulated data**	**Real data**
**PERCENTAGE OF REACTORS TO THE SKIN-TEST (%)**		
Average	8.7	8.5
Range (min–max)	0.0–70	0.08–98.0
3rd Quartile	10.0	9.5
Standard deviation	9.5	12.4
**DURATION OF BREAKDOWN (NO. DAYS)**		
Average	365.9	365.7
Range (min–max)	180.0–1,260	60.0–5,457
3rd Quartile	420.0	409.0
Standard deviation	174.7	395.1
**EPIDEMIOLOGICAL PARAMETERS**		
Rate of external infection (α) [days^−1^]	5 × 10^−7^	
Transmission coefficient (β)	0.012	
Rate from exposed to infectious state (σ) [days^−1^]	0.04	
Rate from infectious to test-sensitive state (γ) [days^−1^]	0.5	
Rate of detection (Ω)	0.60	
**GENETIC PARAMETERS OF SUSCEPTIBILITY**		
**Underlying scale**		
Genetic variance	0.3	
Environmental variance	0.3	
Accuracy of selection	0.63	
**Observed scale**		
Genetic variance	0.0034	0.0032
Phenotypic variance	0.032	0.031
Heritability	0.106	0.103

The rate of progression from the *E* to *I* state, σ, corresponded to an exposed state duration (1/ σ) of 25 days (Table 1). The rate of progression γ from *I* to *T* state suggested that, once a cow becomes infectious, she is expected to respond to the skin test within (1/γ) 2 days.

### Impact of genetic selection on underlying susceptibility

Genetic selection resulted in a reduction in the mean underlying susceptibility to bTB and the corresponding genetic variance (Supplementary Figure 2). The initial underlying susceptibility phenotype in the base population was simulated with a mean of zero, hence the decrease in susceptibility due to selection is depicted by negative values in Supplementary Figure 2B. Greater reduction was observed for higher selection intensities. As expected, no change in genetic variance and mean susceptibility was observed over generations in absence of selection.

### Impact of genetic selection on epidemic profiles

**Figure 2:**
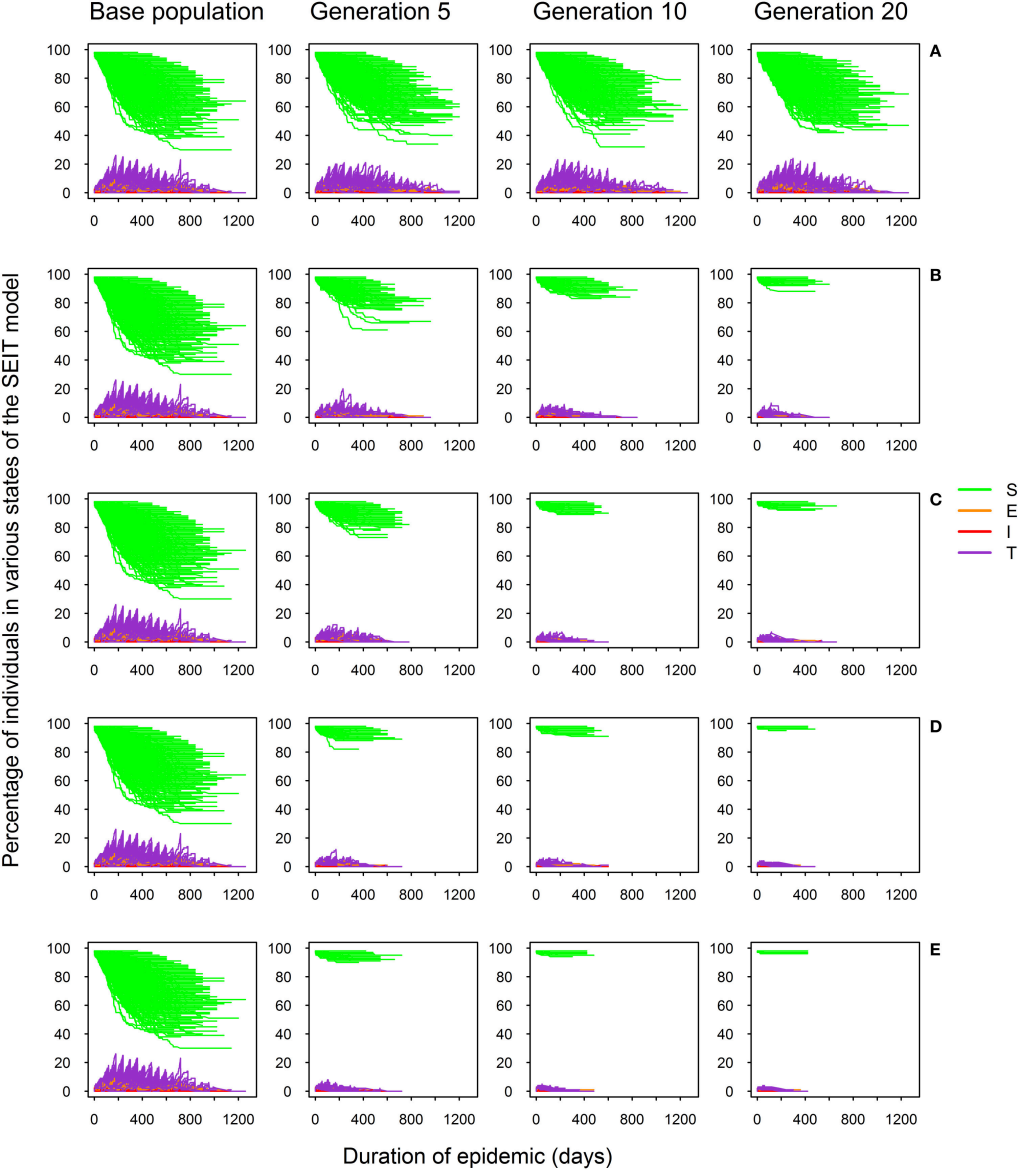
*SEIT* model profiles across 20 generations for five selection intensities defined by the percentage of selected sires: 100% (no selection; **A**), 70% **(B)**, 50% **(C)**, 25% **(D)**, and 10% **(E)**; proportion of susceptible (*S*), exposed (*E*), infectious (*I*), and test-sensitive (*T*) individuals during the course of the epidemic.

Figure 2 shows the *SEIT* profiles (proportions of individuals in different states of the *SEIT* model) over successive generations for different selection intensities. The proportion of infected animals, including those in the exposed, infectious and test-sensitive states, was high before selection and significantly reduced after implementation of selection. As expected, there was no significant reduction in the number of infected individuals and duration of the epidemic over generations when no selection was performed (Figure 2A). Selection noticeably affected both the epidemic risk (illustrated here by the decreasing number of epidemic profiles over successive generations in Figures 2B–E) and severity (illustrated here by the number of infected (*E, T*, and *I* states) individuals and epidemic duration). As expected, the higher the selection intensity, the stronger was the impact on the epidemic profile (Figures 2B–E).

### Impact of genetic selection on risk of a breakdown to occur

**Figure 3:**
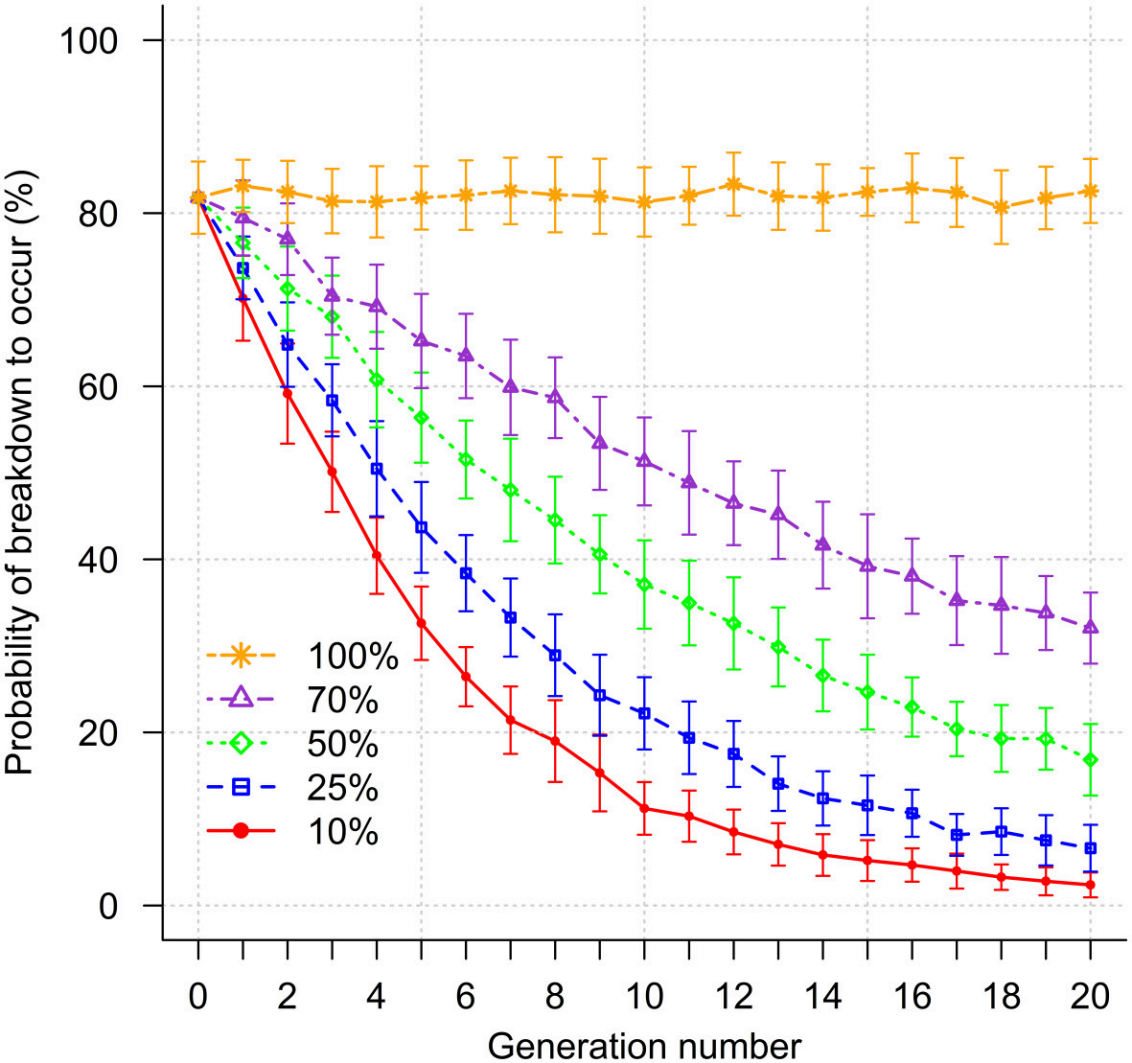
Impact of genetic selection on risk of breakdown (probability of a breakdown to occur). Selection intensities correspond to selection of the 10, 25, 50, 70, and 100% (no selection) most resistant sires.

Figure 3 shows a decrease in the probability of a breakdown occurring with increasing selection intensity. Prior to selection, the mean probability of occurrence of a breakdown was 81.8%. When higher selection intensities were applied corresponding to selection of the 10 and 25% most resistant sires, this probability was halved after 4 and 6 generations, respectively. A similar result was achieved for lower selection intensities (50 and 70% most resistant sires) after 9 and 15 generations, respectively.

### Impact of genetic selection on percentage of secondary cases and duration of their occurrence within breakdowns

**Figure 4:**
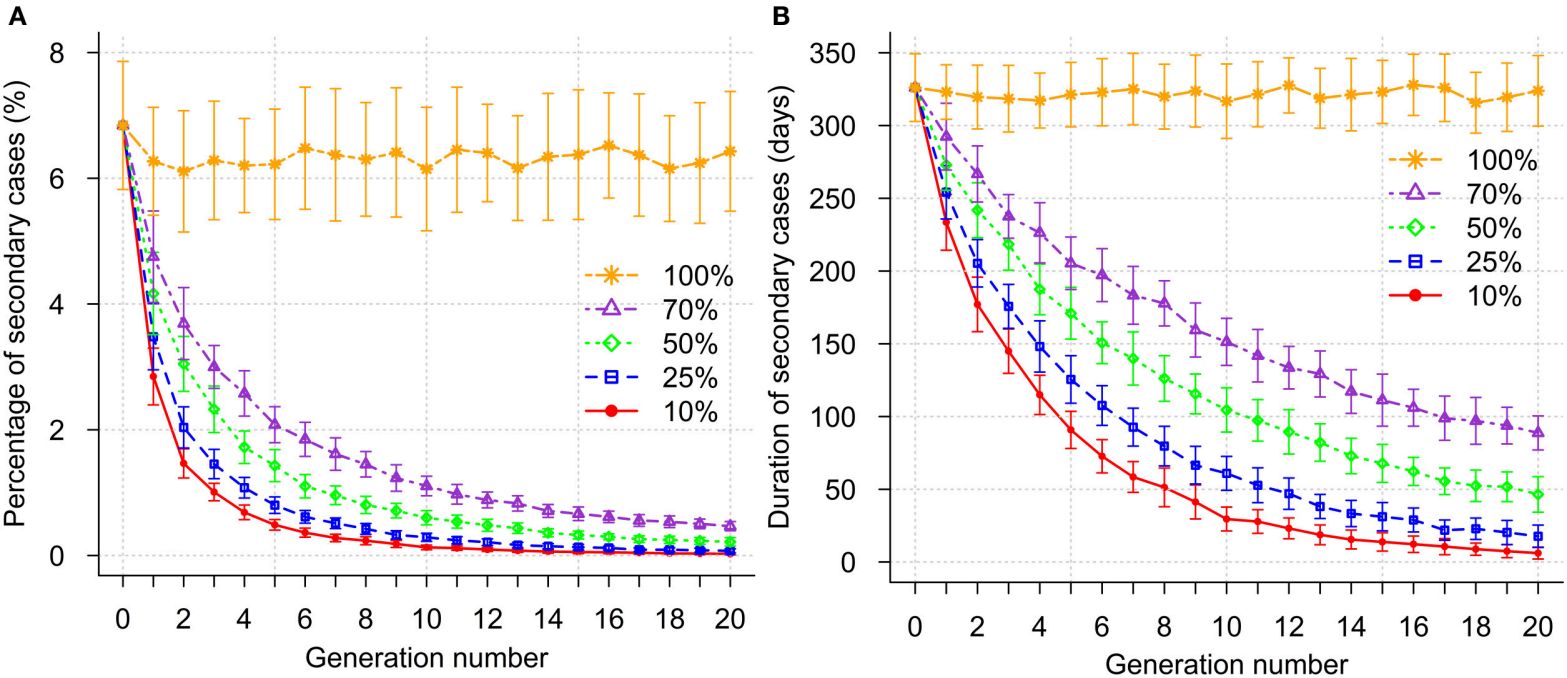
Impact of genetic selection on percentage of secondary cases **(A)** and duration of secondary case occurrence **(B)** within a breakdown. Selection intensities correspond to selection of the 10, 25, 50, 70, and 100% (no selection) most resistant sires.

Genetic selection led to a decline in the percentage of secondary cases per breakdown (Figure 4A). To reduce the percentage of secondary cases per breakdown to <1%, 4, 5, 7, and 11 generations of selection were required when 10, 25, 50, and 70% most resistant sires were selected, respectively. The corresponding duration of secondary case occurrence within a breakdown in these generations was reduced by more than half to 114.9, 125.5, 139.9, and 141.8 days for the four selection intensities, respectively, compared to 326.1 days before selection was introduced (Figure 4B). Furthermore, selection for 12 and 17 generations was required to eliminate the epidemics (occurrence of secondary cases less than or equal to 0.1%) when 10 and 25% most resistant sires were selected, respectively. However, elimination of bTB was not possible with lower selection intensities (greater proportion of sires selected) during the simulated selection period of 20 generations. In the absence of selection, the percentage of secondary cases and time for induction of secondary cases fluctuated around the initial mean (Figures 4A,B).

**Figure 5:**
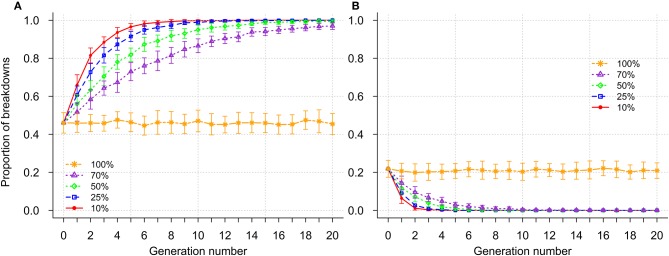
Impact of genetic selection on the percentage of secondary case(s) occurrence within a breakdown; mild (≤3% secondary cases - **A**) and severe (>10% secondary cases - **B**); selection intensities correspond to selection of the 10, 25, 50, 70, and 100% (no selection) most resistant sires.

The effects of genetic selection when breakdowns were categorized according to severity are illustrated in Figure 5 and Supplementary Figure 3. Prior to selection, the proportion of mild, moderate and severe breakdowns was 0.46, 0.32, and 0.22, respectively. During selection, the overall severity of breakdowns decreased across generations (Figure 5). When high selection intensities were applied (selection of the 10 or 25% most resistant sires), almost all breakdowns became mild by generation 10 (Figure 5A). However, it was only when selection of the 10% most resistant sires was implemented that breakdowns became short at the end of selection (Supplementary Figure 3A). Proportion of long breakdowns was reduced by more than 50% after 2, 2, 3, and 4 generations for selection of 10, 25, 50, and 70% most resistant sires, respectively (Supplementary Figure 3B). In the absence of selection, severity of breakdowns remained constant, with slight fluctuations across generations (Figure 5; Supplementary Figure 3).

The above results collectively demonstrate how genetic selection has the potential to reduce the probability of a breakdown occurring and the severity of the breakdowns that do eventually occur.

### Impact of variation in epidemiological parameters

Scenarios with a 10-fold increase in the external rate of infection (α = 5 × 10^−6^ days^−1^ instead of 5 × 10^−7^ days^−1^) are shown in Supplementary Data Sheet 1. All other parameters being the same, this increase led to a small non-significant tendency toward more severe breakdowns in early generations but did not influence the impact of genetic selection on disease epidemic, probability of breakdown occurrence and severity of breakdowns.

The reduction of the skin test sensitivity to 0.30 from 0.60 led to an increase in the severity of breakdowns in terms of number of secondary cases and duration but did not affect the probability of a breakdown to occur (Supplementary Data Sheet 2). Importantly, the impact of genetic selection on the disease transmission dynamics was similarly demonstrable in the case of reduced sensitivity of the skin test.

### Comparison between SEIT and SETI models

The impact of genetic selection on the risk and severity of breakdowns under the two models were very similar (Supplementary Figure 4). For the same parameter values, slightly more secondary cases per breakdown were generated with the *SEIT* (6.8%) compared to the *SETI* (5.8%) model in the base population (unselected population). The same number of generations was required in either model to reduce the probability (risk) of a breakdown to occur by half. Similarly, the difference in time required to achieve a certain percentage of reduction (e.g., 50%) in secondary cases or time for induction of secondary cases between the two models was always less than one generation (Supplementary Figure 4).

## Discussion

Considerable advances in infectious disease control may be achieved by selective breeding programmes that include disease resistance of animals in the breeding goal (44). In this context, a breeding programme that exploits existing genetic variation in host susceptibility to bTB could form an important part of the national bTB eradication strategy (11–13, 15, 39). However, quantitative genetics theory alone cannot predict how genetic gain in disease resistance translates into reduction of bTB breakdown risk and severity. The novelty of the present study lies in (i) the development of a genetic epidemiological model that combines for the first time quantitative genetics and epidemiological dynamics of bTB, and (ii) the ability of this model to assess the consequences of genetic selection for enhanced host resistance on bTB prevalence and dynamics.

Our choice of model parameter values was informed by previous literature estimates (5, 27–31, 45) and bTB field data in order to represent UK field conditions. Similarities between model and field or experimental data are essential for drawing reliable conclusions from model predictions (46). In the present study, real data were somewhat more variable than simulated data as manifested by a wider range and greater standard deviation. Otherwise, the simulated model outputs, including mean values and genetic parameters, were similar to results obtained from field data analysis. The distributions of percentage of reactors to the skin test in both real and simulated data were characteristically skewed to the right and correlated with breakdown duration. Skewness in the distribution of disease traits may be attributed to between animal genetic variation (20) and also environmental effects (47). In the real data, other factors such as differences in herd size, management, badger prevalence and climatic conditions are likely to contribute to the diversity observed in epidemic characteristics (42, 48, 49). Many of these factors are recorded in practice, and can be captured by statistical models and accounted for in the genetic evaluation. Other, non-systematic sources of variation would constitute noise in the statistical models. Increasing model complexity by including various systematic or non-systematic effects into the simulation model may increase variability in the model predictions, but would not affect selection response.

Although the bTB model in the present study differs from previous epidemiological bTB models that did not incorporate genetic variation in the host, the estimated population average transmission coefficient β was within the range of transmission coefficients (0.006–0.014 days^−1^) previously reported (5, 27, 29, 31, 50). The duration of the exposed state (*E*) in our model was 25 days, thus slightly higher than the 20 days estimated by O'Hare et al. (31) using UK data and a *SETI* model. In our study, an animal that became infectious was expected to become detectable within 2 days. This short time interval may be sufficient for some additional infected animals to infect others prior to their own diagnosis and subsequent removal from the herd. This may partly explain the persistence of bTB in the UK despite the on-going regime of skin testing and slaughtering of positive reactors. The 2 days between the *I* and *T* states in the present study is comparable to the 1.8 days estimated by Conlan et al. (5), where early infectiousness was assumed (considering animals in both *E* and *T* states in the *SETI* model to be infectious). In their model the *E* state was referred to as the occult state to denote that, although infectious, animals were not detectable by the skin test (5). These estimates would imply that, once animals are infectious a relatively short time is required before they can be detected by the skin test.

Several important implications arise from our results as far as interpretation of bTB transmission and evaluation of control strategies are concerned, particularly with regards to the implementation of genetic selection for increased host disease resistance. Although the potential of the latter as a complementary strategy for disease control has been recognized (10), its utility in terms of reducing disease risk, prevalence, and severity has not been previously assessed.

Susceptibility on the underlying scale affects the probability of an individual to become infected. Therefore, as animals become more resistant, the expectation is for them to become less likely to be infected. Our results demonstrate how reduction in individual infection probability as a result of genetic selection for host resistance to bTB relates to the probability of breakdowns to happen in the first place. Equally important, even when a breakdown was to occur, it would be less severe in terms of number of infected individuals and duration compared to a no selection scenario. Thus, our results are in agreement with previous studies that demonstrated that selection can reduce both the risk and severity of epidemics for other diseases in livestock and fish (17, 20, 21, 51, 52). This is expected to lead to a reduction, not only in frequency of future breakdowns but also in economic losses, as prolonged breakdowns consume substantial resources. Furthermore, as selection reduces the number of reactors during a bTB breakdown, it is also expected to reduce the risk of recurrence (53, 54). Recurrence has been found to be high in the UK, where 23% (38%) of breakdowns recur within 12 (24) months despite the on-going testing regime (55).

We explored the amount of genetic progress in bTB resistance when sires were selected at different levels of selection intensity. Simulating different selection intensities provides insights into future options for breeders. In all cases, our model predicted that most benefits would emerge within the first 5–10 generations of selection. The lowest selection intensity considered here, corresponding to selection of the 70% genetically most resistant sires, reflects a conservative approach that may be taken by breeders regarding novel traits in the breeding programme (G. Banos, unpublished data available upon request). Our results suggest that with such low selection intensity, genetic selection alone would not eradicate bTB by the time England and Wales are set to achieve OTF status (year 2038, which would correspond to 4–5 generations in conventional breeding programmes or about 2–2.5 generations in genomic breeding programmes). Thus, it would be tempting to consider medium to high selection intensities in the breeding programme. However, care must be taken when higher selection intensities are opted for because of possible antagonistic genetic correlations between bTB and other important dairy traits (56) in the breeding goal. Antagonism would imply that genetically improving one trait compromises the other and may be dealt with using an optimized selection index of multiple traits.

Selection could be applied complementarily to other interventions including existing measures in order to expedite the eradication process. In the context of the genetic-epidemiological model described here, this would include continued efforts to reduce the external source of infection, referring to wildlife-to-cattle, and neighboring and incoming cattle-to-local cattle transmission. Furthermore, improvement of sensitivity of major bTB diagnostic tools such as the skin test and abattoir inspection could translate into an increased removal rate of infected cattle and, hence, reduce the average herd infectivity; further research would be needed to quantify such possible benefits. Other options not included in our model such as selecting for increased resistance in dams in addition to sires, genetic selection to reduce infectivity in addition to susceptibility (57), and genomic selection could also be explored. The latter has a potential to considerably shorten the generation interval and expedite genetic gains (58, 59).

Given the global importance of bTB, a large number of epidemiological models for bTB transmission have been published in the scientific literature (5, 25, 30, 31, 45, 50, 60). The models differ widely in their scope and purpose, although the majority of models focus on estimating transmission parameters and transmission routes from epidemiological data, or explore the impact of different surveillance or control options on bTB prevalence. To the best of our knowledge, this is the first model that incorporates genetic disease control strategies.

To model within-herd transmission dynamics, the epidemiological bTB model in the present study adopted a similar compartmental approach as in recently published stochastic epidemiological bTB models that have been fitted to UK bTB data (5, 30, 31). However, to assess the impact of genetic selection on bTB prevalence and dynamics, we adopted the *SEIT* transmission model, while a more optimistic *SETI* model in terms of transmission has been previously used in the majority of epidemiological studies. Information about the suitability of *SEIT* or *SETI* models for bovine tuberculosis is non-existent. In other diseases, both *SETI* and *SEIT* models have proven to be biologically reasonable. Diseases in humans such as HIV or hepatitis C show epidemiological processes concordant with the *SEIT* model, with window periods between infection and detection when the infected individuals are also infectious (61). Furthermore, in case of human tuberculosis, the window period for the Mantoux test (a skin test based in the presence of immune response against tuberculin) is between 2 and 6 weeks (62), with an incubation period for the disease of 2–12 weeks, thus potentially allowing enough time for individuals to become infectious before the window period closes. This is particularly true when the individual has a slow immune response that delays detection. While the onset of infectiousness in relation to reactivity to the skin test is currently not known, inference based approaches have demonstrated an equally good model fit to empirical data if cattle were assumed to become infectious without epidemiological latency, i.e., before entering the detectable state (5). Results from the present study demonstrated that the *SEIT* model indeed represented the “worst” case scenario resulting in more secondary cases per breakdown than the *SETI* model. The number of secondary cases increased in the *SEIT* model because animals became infectious and could infect others before being detected and removed. However, despite the difference between the models in terms of bTB transmission, the present study showed that the impact of genetic selection tended not to differ much between the two models. The similarity between the models may be partly attributed to the relatively short time interval of 2 days estimated between the *I* and *T* states. Differences between the model predictions might have been more pronounced if this time interval was longer and the contribution of the external force of infection (α) higher.

Some important assumptions warrant further discussion. In the present study, the external source of infection (α) was kept constant across generations. However, selection is expected to reduce external infection because as animals become more resistant and the number of infectious cows declines, cattle-to-cattle and cattle-to-wildlife-to-cattle transmissions are expected to reduce over time. Therefore, keeping the external source of infection constant in the simulations depicts a somewhat conservative approach regarding the favorable impact of genetic selection. Similarly, the accuracy of selection was kept constant in the simulations, but may also decline as bTB outbreaks decrease across generations and genetically resistant cows become harder to identify. Lower accuracies could slow down response to selection. However, continuous bTB field data collection combined with optimized bTB genetic evaluation methods would counter the effect of reduction in disease prevalence and maintain accuracy of selection over generations. A common concern about genetic control strategies is the impact of selection for host resistance on potential pathogen evolution, which may slow down the predicted genetic gain in host resistance. However, in the case of bTB, the relatively low genetic variability of *M. bovis strains* within cattle populations (63), combined with the evidence from quantitative genetics studies incorporated in the model that host resistance is controlled by many genes, implies that this risk can be considered as negligible (64).

Even though the model aimed to mimic the overall population structure of UK dairy herds, demographic characteristics were not explicitly included in the present study. Not including specific demographic characteristics would particularly affect the estimates of breakdown risk, which are conditional on the introduction of infected cows in each herd. It should be noted that whilst these estimates are useful means to quantify and compare selection response, they differ from the absolute risk of a bTB breakdown, which also depends on the probability of index cases to occur in the first place and on various additional factors not considered in the model, such as cattle movement across herds of different sizes, or different management characteristics and exposure to wildlife (40, 42, 49).

Furthermore, the parameters used in the present study were obtained from literature estimates and statistical comparison of simulated with real disease data. Whilst this approach is very common for epidemiological prediction models (20, 21, 23, 25, 27), it cannot be guaranteed that alternative sets of parameter values would not provide a better model fit to the data. To test this, more sophisticated statistical inference techniques (30, 31, 37) would be required. Thus, future modeling studies may build on our work, including explicit descriptions of additional risk factors associated with bTB prevalence combined with statistical inference techniques for parameter estimation.

Apart from genetic variation in cattle resistance to bTB, no other sources of genetic or individual variation in the model parameters were included in the model. This is in line with standard animal breeding approaches, which focus primarily on selection for disease resistance. Although it is possible that cows may also vary genetically in the duration of the exposed or infectious state, or even in their skin test sensitivity, including genetic variation in the corresponding epidemiological model parameters may affect epidemiological characteristics within each generation (19, 22), but will not affect the predicted responses to selection for disease resistance. Also, within the context of the above assumptions, changing the values of some key epidemiological parameters did not seem to affect the impact of genetic selection on disease transmission dynamics manifested by probability of breakdown occurrence and severity of breakdowns. However, these parameters would largely determine the dynamics of a bTB epidemic, especially when genetic selection is not taken into account. Specifically, our analyses revealed that a decreased sensitivity of the skin test would lead to more severe breakdowns, affecting both the number of secondary cases and the duration of breakdowns. Therefore, the development of diagnostics with high sensitivity that would allow early and accurate detection of infected individual is strongly encouraged.

In the present study, the purpose of some simplifications was to allow a clear demonstration of the predicted effects of genetic selection for enhanced host resistance against the disease on the evolution and dynamics of epidemics. We maintain that the predicted impact of selection is still relevant when such simplifications are lifted. For example, we assumed that all herds in the simulation had the same size, which was similar to the average herd size in the UK dairy cattle population. In reality, herd size varies implying possibly different individual profiles of epidemics in larger vs. smaller herds. However, at population level, the overall epidemic profile will reflect that of the average-sized herd. Furthermore, sire distribution across herds is independent of herd size meaning the overall accuracy of genetic evaluation and selection would not be very close to what was simulated here.

The genetic-epidemiological model developed in the present study provides the first quantitative estimates of the impact of selection for increased resistance on bTB prevalence. In all cases, selection for increased resistance translates into noticeable epidemiological benefits. Strong selection intensities on bTB resistance would particularly benefit high risk geographic areas where the disease is highly prevalent and highly resistant sires are required. The prospects of assimilating bTB resistance into the national selection programme are convincing despite the moderate heritability of the trait. For example, while heritability of clinical mastitis in dairy cattle is low and unfavorably correlated with milk production traits, mastitis is nonetheless included in selective breeding programmes in several countries (65, 66).

## Conclusions

We developed a genetic epidemiological model to investigate the impact of genetic selection for enhanced bTB resistance on disease prevalence and dynamics. Results demonstrated that genetic selection could substantially reduce bTB prevalence and severity of breakdowns over generations of selection. Our study also highlights the importance of considering genetic selection as an additional control tool that can complement existing strategies. Considering genetic selection is pertinent, especially with the view of accelerating the control and eradication of bTB to achieve the national goal of OTF status by 2038 as planned in England and Wales. Future work could consider additional genetic selection strategies such as selection for resistant dams and selection for reduced individual animal infectivity.

## Availability of data

Data generated from the present study will be made available on request to qualified researchers.

## Author contributions

KR, AD-W, EG, JW, and GB designed the study. KR and ES-M performed the analysis. KR, ES-M, ST, OA, AD-W, and GB interpreted the results. KR prepared the manuscript. KR, ES-M, ST, OA, EG, JW, AD-W, and GB revised the manuscript and improved its content.

### Conflict of interest statement

The authors declare that the research was conducted in the absence of any commercial or financial relationships that could be construed as a potential conflict of interest.

## Supplementary material

Supplementary Data Sheet 1 Simulated scenario with increased rate of external infection (α = 5 × 10^−6^ instead of 5 × 10^−7^), all other parameters remaining constant. Figures shown are: *SEIT* model profiles across 20 generations for five selection intensities defined by the percentage of selected sires: 100% (no selection; **A**), 70% **(B)**, 50% **(C)**, 25% **(D)**, and 10% **(E)**; proportion of susceptible (*S*), exposed (*E*), infectious (*I*), and test-sensitive (*T*) individuals during the course of the epidemic. Impact of genetic selection on risk of breakdown (probability of a breakdown to occur). Selection intensities correspond to selection of the 10, 25, 50, 70, and 100% (no selection) most resistant sires. Impact of genetic selection on percentage of secondary cases **(A)** and duration of secondary case occurrence **(B)** within a breakdown. Selection intensities correspond to selection of the 10, 25, 50, 70 and 100% (no selection) most resistant sires. Impact of genetic selection on the percentage of secondary case(s) occurrence within a breakdown; mild (≤3% secondary cases—**A**) and severe (>10% secondary cases—**B**); selection intensities correspond to selection of the 10, 25, 50, 70, and 100% (no selection) most resistant sires. Impact of genetic selection on the duration of secondary case(s) within a breakdown; short (≤180 days - **A**) and long (>365 days - **B**); selection intensities correspond to selection of the 10, 25, 50, 70, and 100% (no selection) most resistant sires.

Supplementary Data Sheet 2 Simulated scenario with decreased sensitivity of the skin test (0.30 instead of 0.60), all other parameters remaining constant. Figures shown are: *SEIT* model profiles across 20 generations for five selection intensities defined by the percentage of selected sires: 100% (no selection; **A**), 70% **(B)**, 50% **(C)**, 25% **(D)**, and 10% **(E)**; proportion of susceptible (*S*), exposed (*E*), infectious (*I*), and test-sensitive (*T*) individuals during the course of the epidemic. Impact of genetic selection on risk of breakdown (probability of a breakdown to occur). Selection intensities correspond to selection of the 10, 25, 50, 70, and 100% (no selection) most resistant sires. Impact of genetic selection on percentage of secondary cases **(A)** and duration of secondary case occurrence **(B)** within a breakdown. Selection intensities correspond to selection of the 10, 25, 50, 70, and 100% (no selection) most resistant sires. Impact of genetic selection on the percentage of secondary case(s) occurrence within a breakdown; mild (≤3% secondary cases - **A**) and severe (>10% secondary cases - **B**); selection intensities correspond to selection of the 10, 25, 50, 70, and 100% (no selection) most resistant sires. Impact of genetic selection on the duration of secondary case(s) within a breakdown; short (≤180 days - **A**) and long (>365 days - **B**); selection intensities correspond to selection of the 10, 25, 50, 70, and 100% (no selection) most resistant sires.

Supplementary Figure 1 Scheme of the compartmental genetic-epidemiological bTB model. The compartments depict the transition between different animal disease states [Susceptible, Exposed (latent), Infectious and Test-sensitive (detectable)] in the adopted *SEIT* model with assumed heterogeneity in underlying host susceptibility to bTB. Once cows in the Test-sensitive state are diagnosed, they are removed from the herd (Removed compartment). The transition between the compartments depends on the background infection (B), the population average values for the epidemiological parameters: transmission coefficient, β; rate of infection from external sources, α; force of infection from herd-mates, λ; progression rate from Exposed to Infectious state, σ; progression rate from Infectious to Test-sensitive state, γ; skin test sensitivity, Ω; and the distribution of the underlying susceptibility of cattle to bTB (g). Genetic selection affects the g and, thus the individual and average rates of progression from Susceptible to the subsequent states.

Supplementary Figure 2 Impact of genetic selection on the host underlying susceptibility to bovine tuberculosis. Changes in genetic variation **(A)** and mean susceptibility on the underlying scale **(B)**; selection intensities correspond to selection of the 10, 25, 50, 70, and 100% (no selection) most resistant of sires.

Supplementary Figure 3 Impact of genetic selection on the duration of breakdown; short (≤180 days - **A**) and long (>365 days - **B**); selection intensities correspond to selection of the 10, 25, 50, 70, and 100% (no selection) most resistant sires.

Supplementary Figure 4 Impact of genetic selection on average risk of breakdown, and percentage and duration of secondary case(s) occurrence within breakdown in the *SEIT* **(A)** and *SETI* **(B)** models. Selection intensities correspond to selection of the 10, 25, 50, 70, and 100% (no selection) most resistant sires. The dashed horizontal lines represent reduction by 50%.
