# Supplementary figures and images for: Impact of Genetic Selection for Increased Cattle Resistance to Bovine Tuberculosis on Disease Transmission Dynamics
Source: Front Vet Sci. 2018 Oct 1;5:237. doi: 10.3389/fvets.2018.00237 (PMC6174293; doi:10.3389/fvets.2018.00237)

**
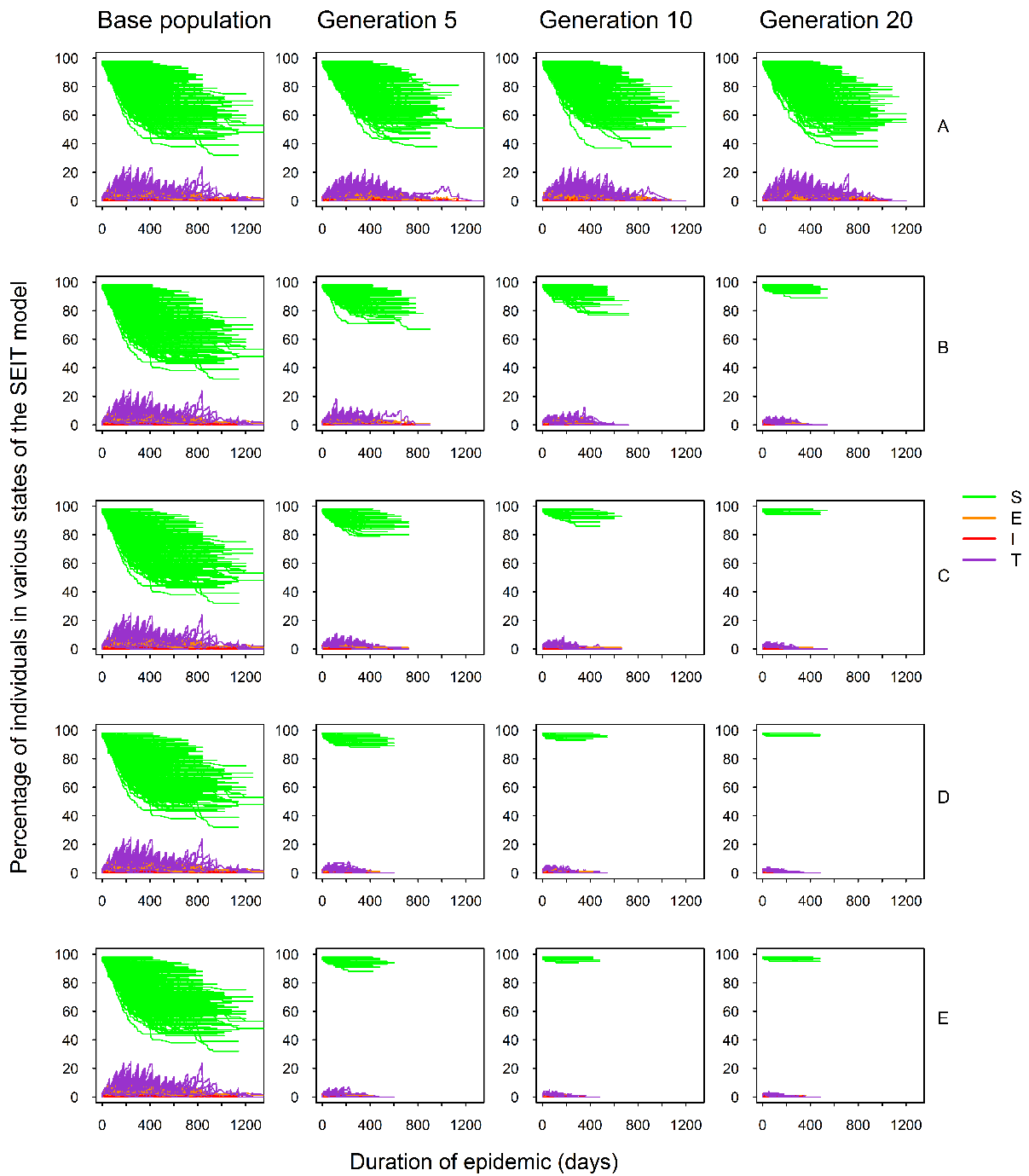
1

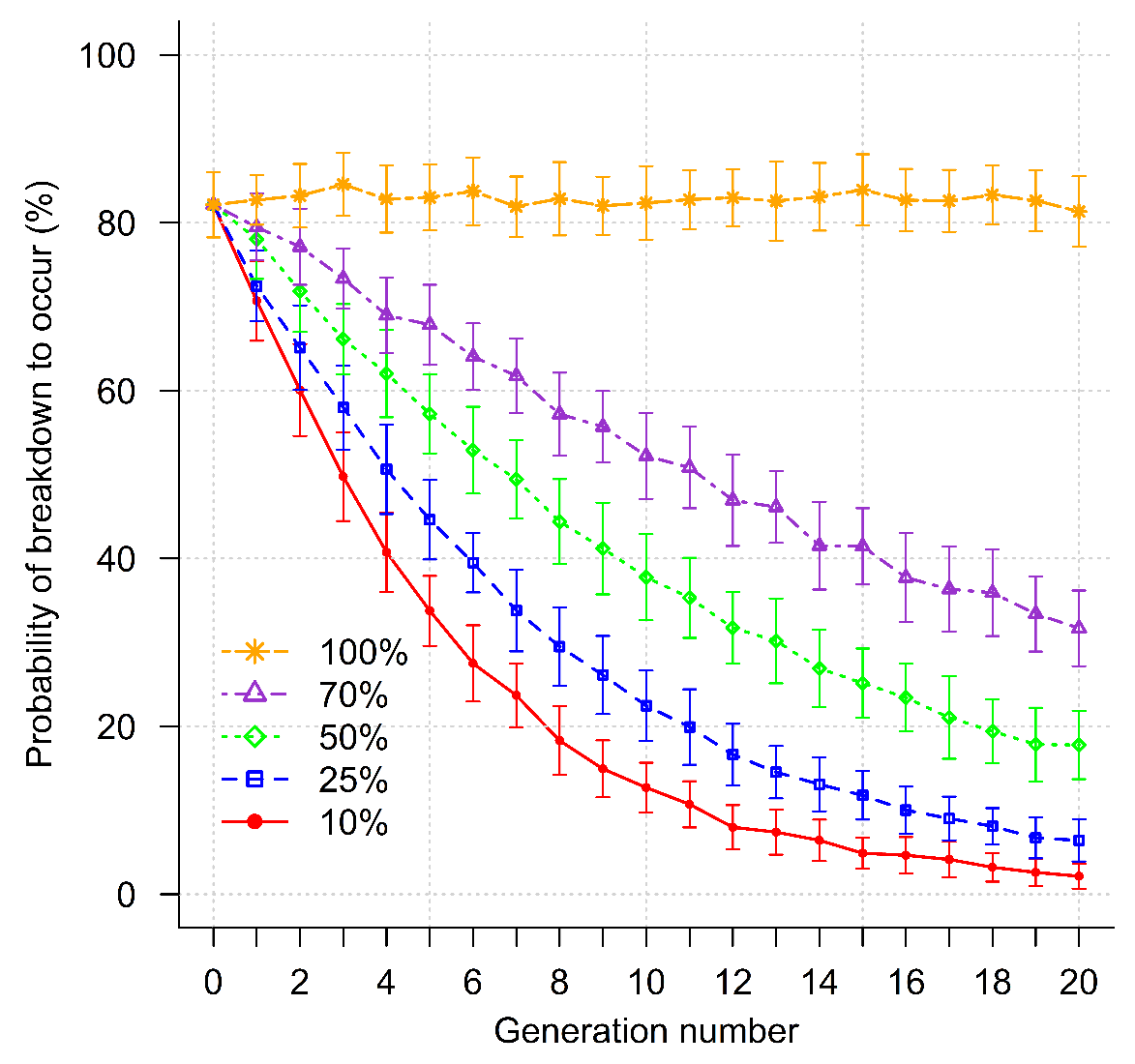
2**

**
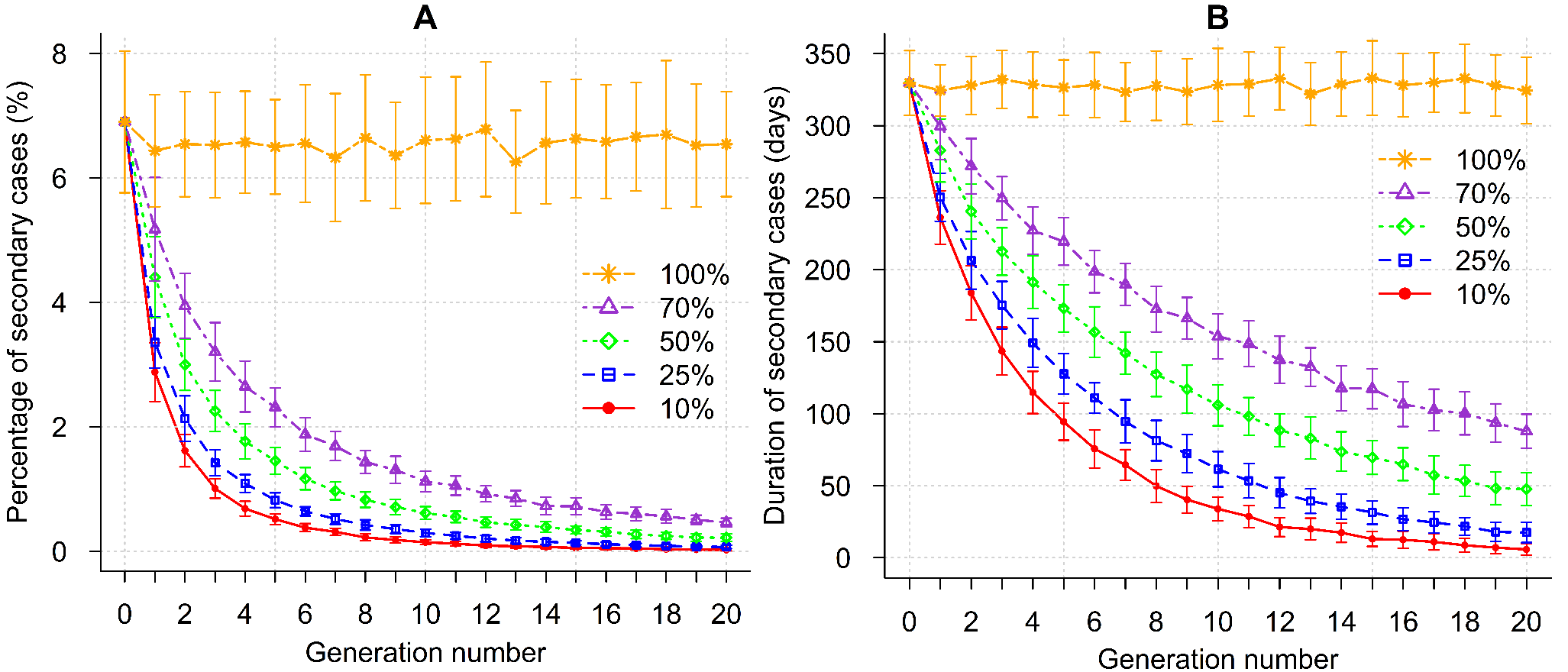
3**

**
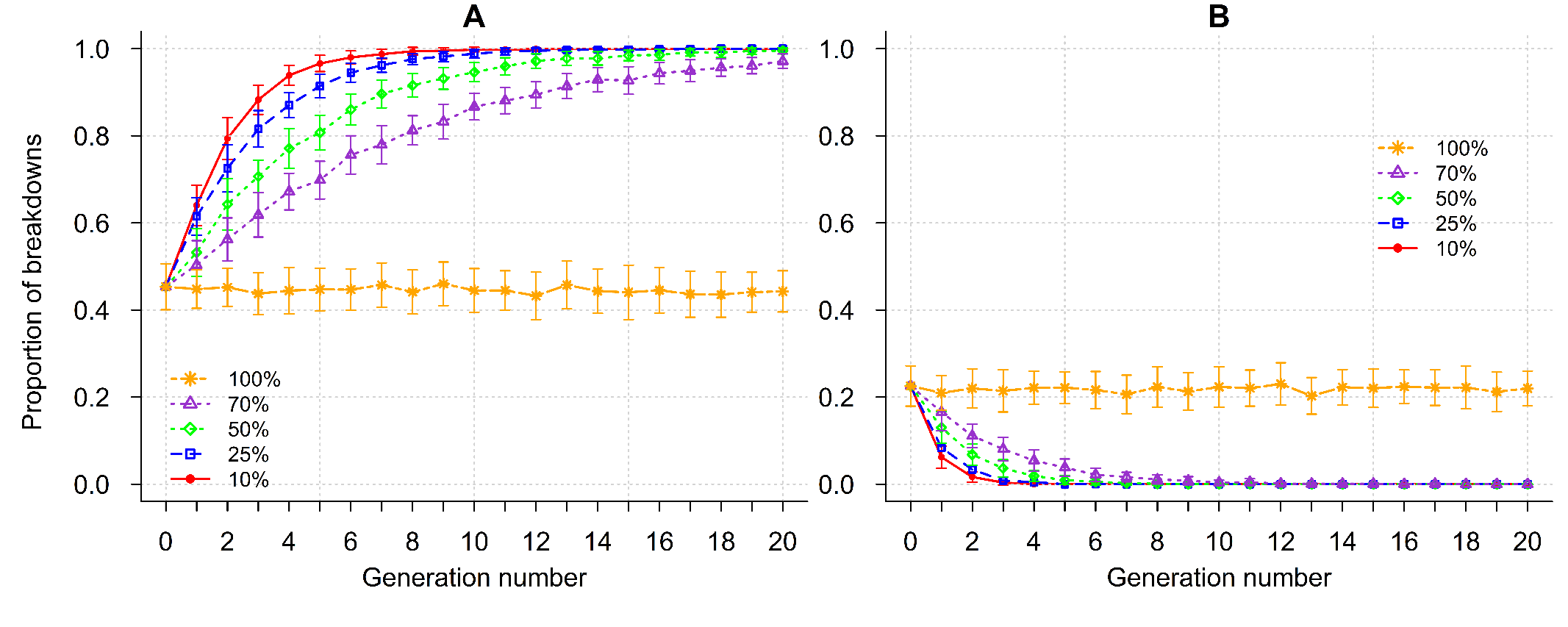
4**

**5**

**
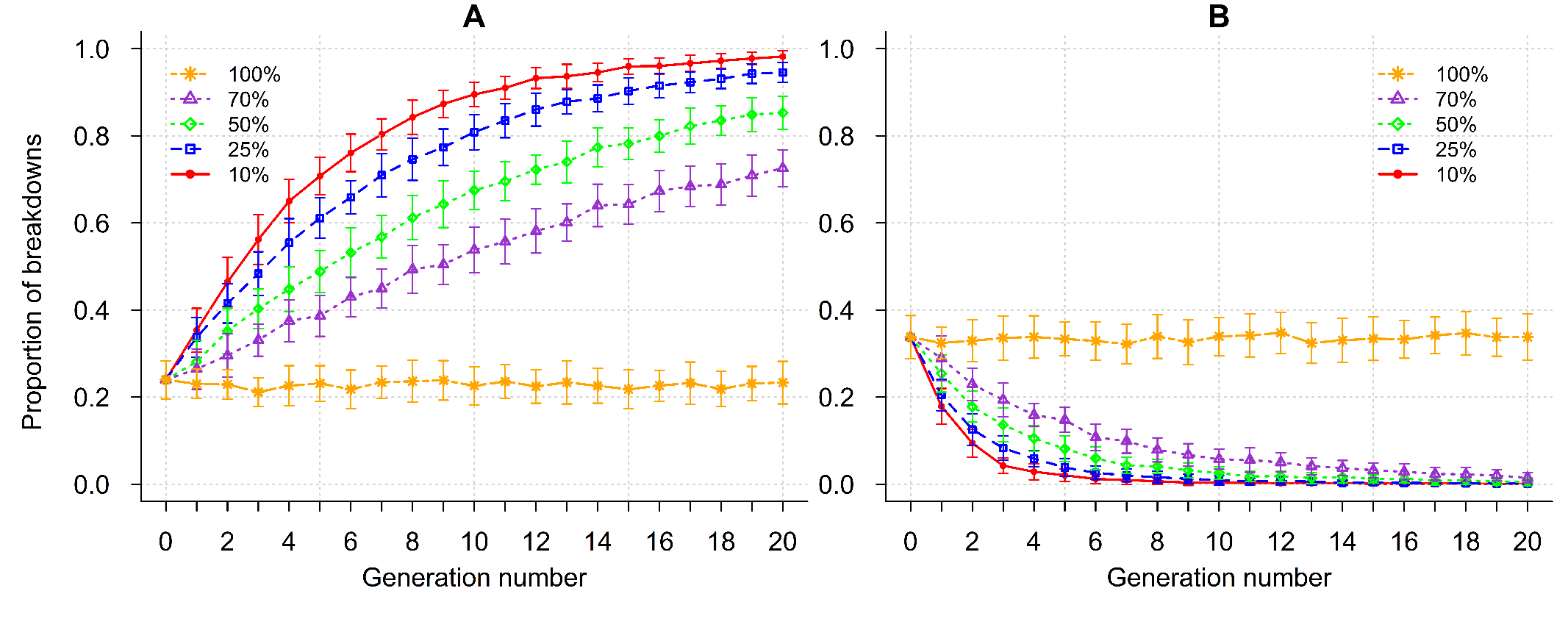
**

Supplement: Supplementary Data Sheet 1 — Simulated scenario with increased rate of external infection (α = 5 × 10^−6^ instead of 5 × 10^−7^), all other parameters remaining constant. Figures shown are: SEIT model profiles across 20 generations for five selection intensities defined by the percentage of selected sires: 100% (no selection; A), 70% (B), 50% (C), 25% (D), and 10% (E); proportion of susceptible (S), exposed (E), infectious (I), and test-sensitive (T) individuals during the course of the epidemic. Impact of genetic selection on risk of breakdown (probability of a breakdown to occur). Selection intensities correspond to selection of the 10, 25, 50, 70, and 100% (no selection) most resistant sires. Impact of genetic selection on percentage of secondary cases (A) and duration of secondary case occurrence (B) within a breakdown. Selection intensities correspond to selection of the 10, 25, 50, 70 and 100% (no selection) most resistant sires. Impact of genetic selection on the percentage of secondary case(s) occurrence within a breakdown; mild (≤3% secondary cases—A) and severe (>10% secondary cases—B); selection intensities correspond to selection of the 10, 25, 50, 70, and 100% (no selection) most resistant sires. Impact of genetic selection on the duration of secondary case(s) within a breakdown; short (≤180 days - A) and long (>365 days - B); selection intensities correspond to selection of the 10, 25, 50, 70, and 100% (no selection) most resistant sires. [file Data_Sheet_1.docx]

**1
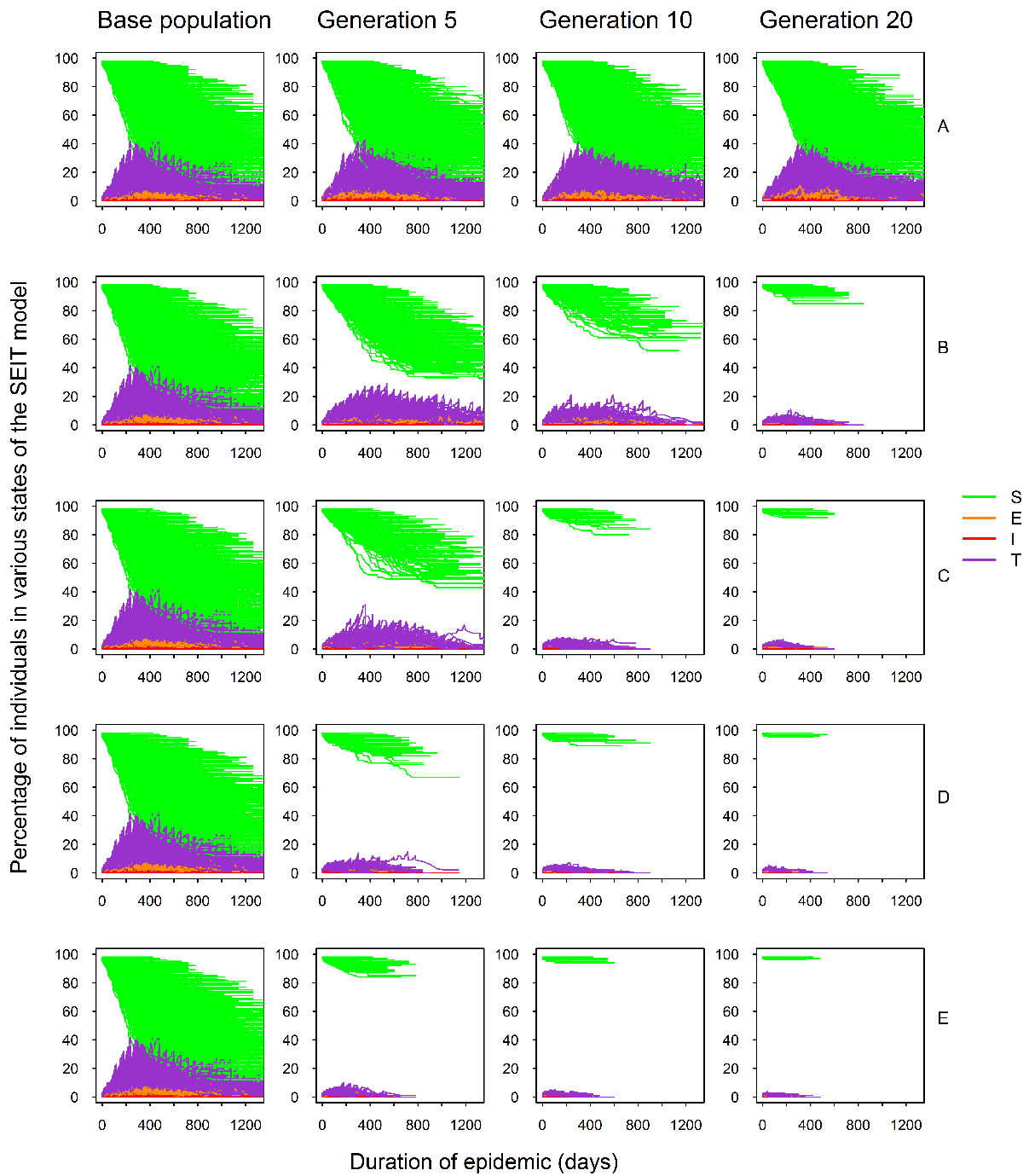

2**


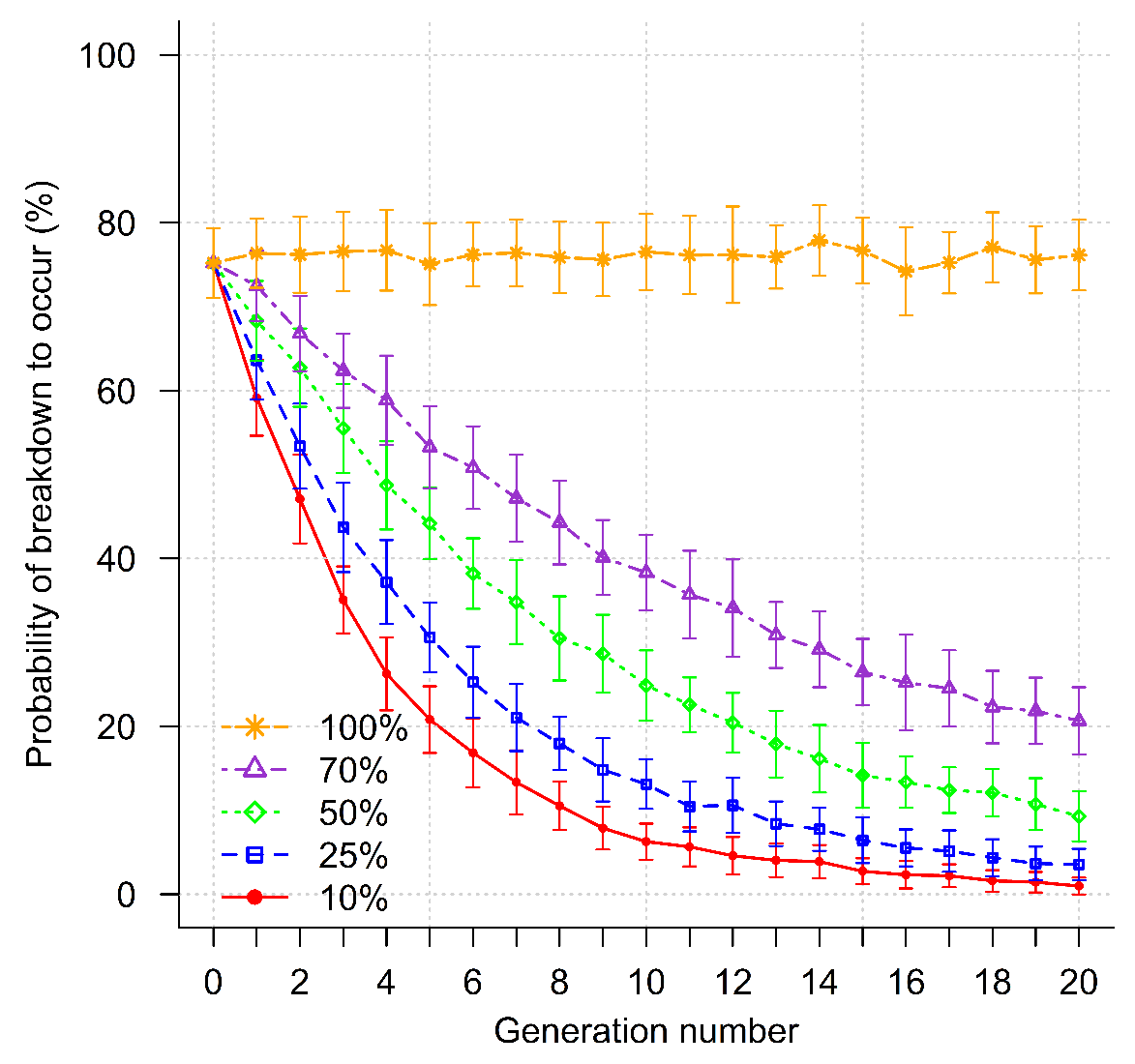


**3**

**
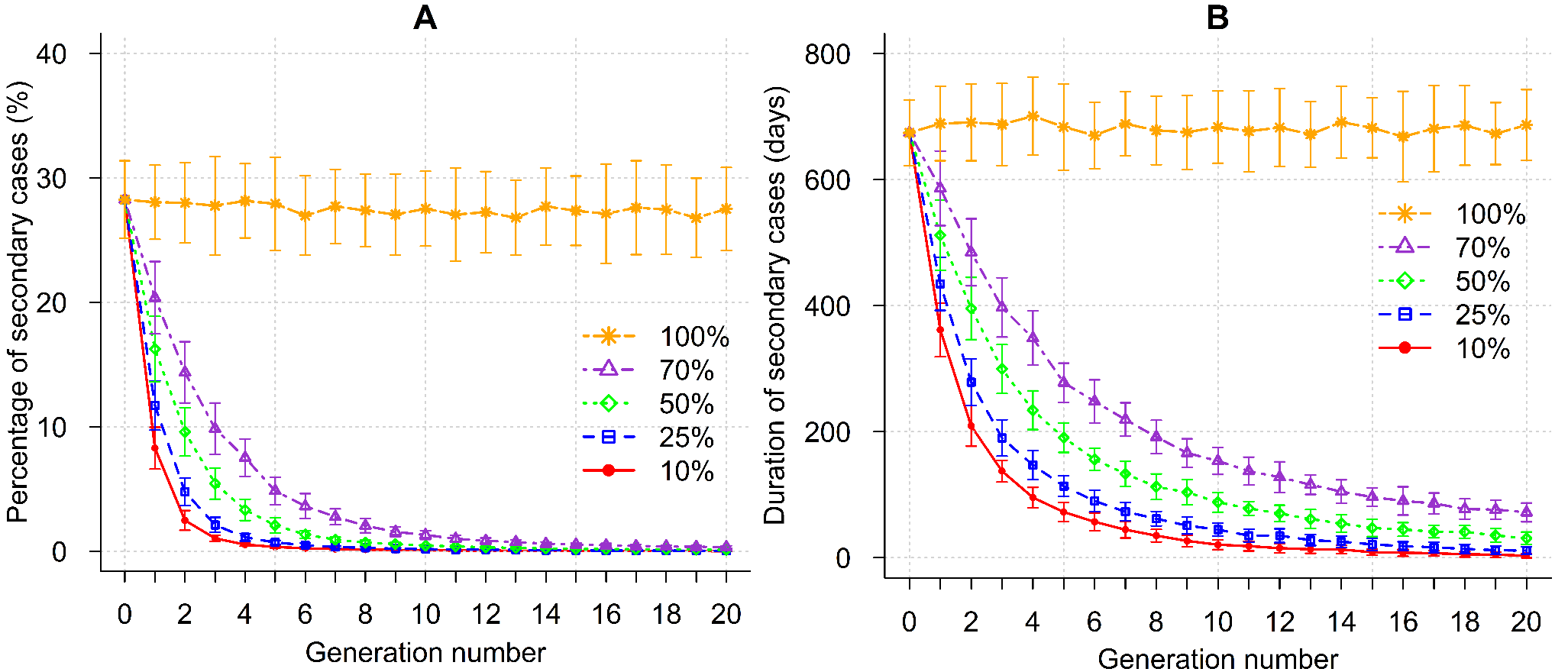
**

**4**


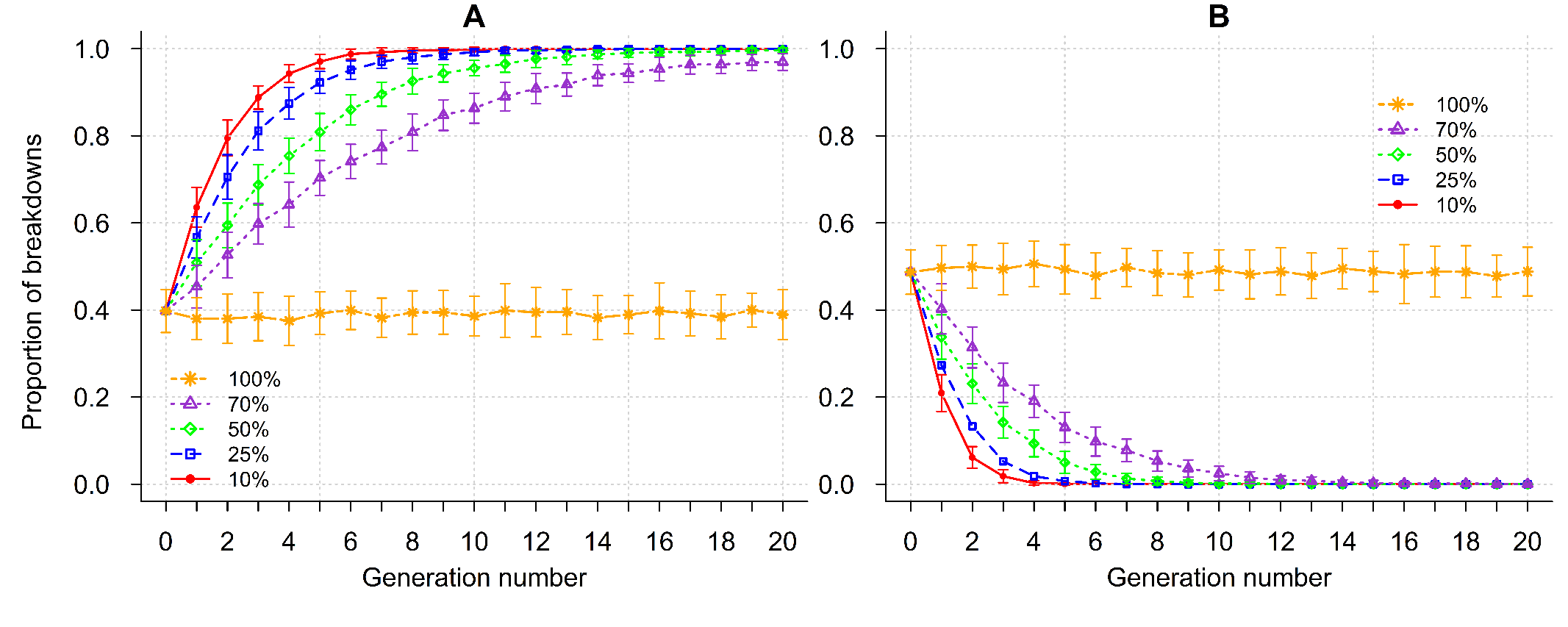


**5**


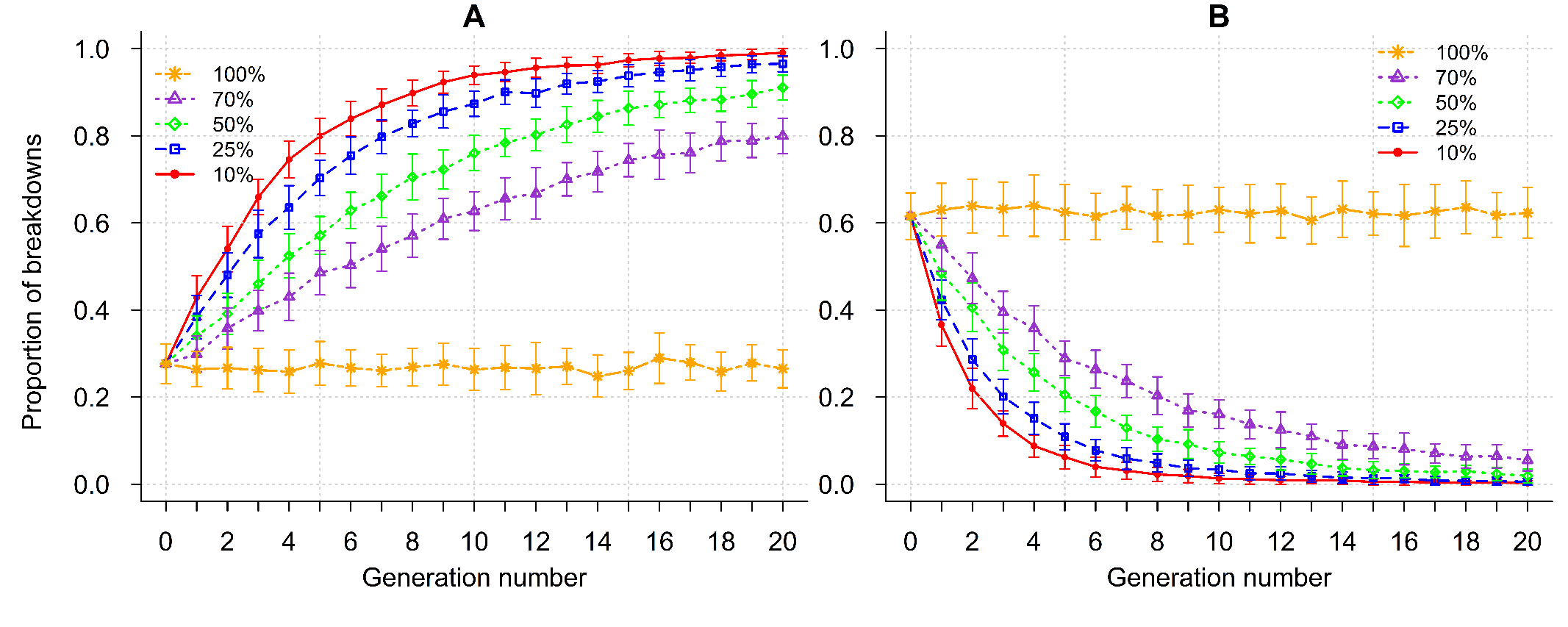

Supplement: Supplementary Data Sheet 2 — Simulated scenario with decreased sensitivity of the skin test (0.30 instead of 0.60), all other parameters remaining constant. Figures shown are: SEIT model profiles across 20 generations for five selection intensities defined by the percentage of selected sires: 100% (no selection; A), 70% (B), 50% (C), 25% (D), and 10% (E); proportion of susceptible (S), exposed (E), infectious (I), and test-sensitive (T) individuals during the course of the epidemic. Impact of genetic selection on risk of breakdown (probability of a breakdown to occur). Selection intensities correspond to selection of the 10, 25, 50, 70, and 100% (no selection) most resistant sires. Impact of genetic selection on percentage of secondary cases (A) and duration of secondary case occurrence (B) within a breakdown. Selection intensities correspond to selection of the 10, 25, 50, 70, and 100% (no selection) most resistant sires. Impact of genetic selection on the percentage of secondary case(s) occurrence within a breakdown; mild (≤3% secondary cases - A) and severe (>10% secondary cases - B); selection intensities correspond to selection of the 10, 25, 50, 70, and 100% (no selection) most resistant sires. Impact of genetic selection on the duration of secondary case(s) within a breakdown; short (≤180 days - A) and long (>365 days - B); selection intensities correspond to selection of the 10, 25, 50, 70, and 100% (no selection) most resistant sires. [file Data_Sheet_2.docx]

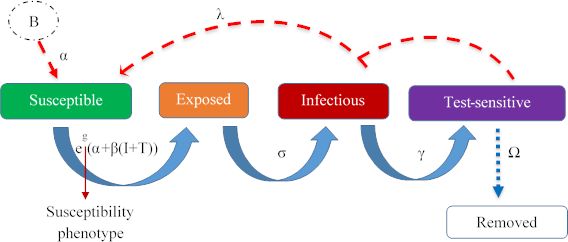

Supplement: Supplementary Figure 1 — Scheme of the compartmental genetic-epidemiological bTB model. The compartments depict the transition between different animal disease states [Susceptible, Exposed (latent), Infectious and Test-sensitive (detectable)] in the adopted SEIT model with assumed heterogeneity in underlying host susceptibility to bTB. Once cows in the Test-sensitive state are diagnosed, they are removed from the herd (Removed compartment). The transition between the compartments depends on the background infection (B), the population average values for the epidemiological parameters: transmission coefficient, β; rate of infection from external sources, α; force of infection from herd-mates, λ; progression rate from Exposed to Infectious state, σ; progression rate from Infectious to Test-sensitive state, γ; skin test sensitivity, Ω; and the distribution of the underlying susceptibility of cattle to bTB (g). Genetic selection affects the g and, thus the individual and average rates of progression from Susceptible to the subsequent states. [file Image_1.TIF]
